# Factors affecting the gut microbiota of rural residents under different physical activity levels: a cross-sectional study

**DOI:** 10.3389/fmicb.2026.1857703

**Published:** 2026-06-15

**Authors:** Zhixing Peng, Fantao Fang, Yanyan Wang, Yonglin Chen, Keer Yang, Yishuo Zhang, Wenzhuo Wang, Biao Song, Weiwei Zhang, Xiaohui Hou, Yu-Heng Mao

**Affiliations:** 1 Guangdong Key Laboratory of Human Sports Performance Science, School of Sports and Health, Guangzhou Sport University, Guangzhou, China; 2 School of Physical Education, Guangzhou Sport University, Guangzhou, China; 3 Guangzhou Center for Disease Control and Prevention (Guangzhou Health Supervision Institute), Guangzhou, China

**Keywords:** BMI, gut microbiota, physical activity, rural residents, sex

## Abstract

**Background:**

Physical activity levels modulate the gut microbiota and thereby influence health status. However, few studies have focused on rural populations. This study aimed to identify the factors influencing the gut microbiota of rural residents under different physical activity levels.

**Methods:**

A total of 311 rural residents (range: 18–90 years) were included in this cross-sectional study. Physical activity levels were assessed using the International Physical Activity Questionnaire (IPAQ). Gut microbial composition was evaluated via 16S rRNA gene sequencing. Spearman’s rank correlation coefficient was used to examine the associations between gut microbiota and physical activity level, age, Body mass index (BMI), and sex. Multiple regression analyses were also performed to further explore these associations.

**Results:**

Among the surveyed rural residents, 9.97% engaged in low-intensity physical activity, 23% in moderate-intensity physical activity, and 67% in high-intensity physical activity. Across different levels of physical activity, age was the most broadly influential variable affecting the microbiota. Sex and smoking/alcohol habits had significant effects on specific genera. BMI was positively correlated with *Escherichia_Shigella* (*r* = 0.192, *p* < 0.001) and negatively correlated with *Bacteroides* (*r* = −0.121, *p* = 0.033). After adjusting for covariates in multiple regression analysis, *Blautia* [B (95% CI): −0.002 (−0.003, −0.001)] was negatively associated with age, *Collinsella* [B (95% CI): −0.015 (−0.027, −0.004)] was negatively associated with sex, and *Segatella* [B (95% CI): 0.000005 (0.0000006, 0.0000009)] was positively associated with physical activity level. Although Spearman correlation and multiple linear regression analyses revealed that the relative abundances of a few bacterial genera were positively or negatively correlated with physical activity level, all effect sizes were very small.

**Conclusion:**

Our study indicated that there were no differences in gut microbiota diversity across different physical activity levels, and only specific genera were associated with physical activity level. Age was the most important factor influencing the gut microbiota. In addition, sex, BMI, smoking, alcohol consumption, and pesticide exposure were also significantly associated with gut microbiota composition. However, the LPA group was composed predominantly of males (93.5%), and the effects observed in the LPA group were essentially limited to males.

## 1 Introduction

As a significant residential population globally, the health status of rural residents not only directly affects individual labor capacity and quality of life but is also closely related to the sustainability of agricultural production and the rural public health system (Jensen et al., 2020; Liu et al., 2025). The lifestyle patterns of rural residents have unique characteristics, their daily physical activity often includes not only voluntary exercise but also a substantial amount of agricultural labor for production purposes, with intensity, frequency, and patterns significantly different from those of urban populations, often accompanied by distinct dietary structures and daily routines. Agricultural labor typically involves prolonged moderate-to-high-intensity physical exertion and often occurs under specific environmental exposures, such as pesticides, fertilizers, and soil microorganisms (Mesnage et al., 2022). These environmental factors have been shown to potentially exert direct or indirect effects on the gut microbiota and may even pose potential health risks (Yang et al., 2026). Such exogenous substances may influence the host gut microbiota through pathways such as the microbiota–gut–brain axis (Sharma et al., 2023).

The gut microbiota, as the largest microbial ecosystem in the human body, plays a vital role in host nutrient metabolism (Schoeler and Caesar, 2019; Zmora et al., 2019), immune regulation (O'Riordan et al., 2025), pathogen defense (Woelfel et al., 2024), and central nervous system function (Peng et al., 2025). Recent studies have shown that physical activity (PA) significantly influences microbial community structure by altering the intestinal microenvironment (e.g., short-chain fatty acids (SCFAs) metabolism, inflammatory cytokine levels), and this association may vary depending on activity intensity and host physiological status. For example, moderate physical activity can improve intestinal barrier function by promoting intestinal motility (Katagiri et al., 2025) and increasing the abundance of SCFAs-producing bacteria (Allen et al., 2018; Batacan et al., 2017). In contrast, chronic overexertion may negatively affect gut microbiota homeostasis by inducing oxidative stress, chronic inflammation, and energy metabolism imbalance (Mach and Fuster-Botella, 2016). Sedentary behavior is associated with the expansion of pro-inflammatory bacteria such as *Escherichia coli* (Castellanos et al., 2020).

However, most existing studies on the relationship between physical activity levels and gut microbiota have focused on urban populations or professional athletes, whose forms of exercise are predominantly gym training or running (Urban et al., 2024; Yang et al., 2024). Research on the relationship between physical activity and gut microbiota in the specific group of rural residents is very limited (Dorelli et al., 2021; Hughes and Holscher, 2021; Wegierska et al., 2022). Simply extrapolating conclusions from urban populations to rural residents may confound the distinct effects of “labor” versus “exercise” on the gut microbiota, potentially overlook critical confounding factors, and even lead to biased conclusions.

Therefore, we conducted a cross-sectional study among rural residents in Guangzhou, China, aiming to characterize the gut microbiota composition of Chinese rural residents under different physical activity levels. Through correlation analysis and multiple linear regression analysis, we sought to explore the factors influencing the gut microbiota of rural residents across varying physical activity levels, thereby providing a scientific basis for the future development of precise health intervention strategies and chronic disease prevention strategies tailored to rural residents with different activity patterns.

## 2 Methods

### 2.1 Study population and data collection

This study adopted a cross-sectional design. Although Guangzhou, as a megacity in southern China, exhibited a high degree of urbanization and modernization overall, after a thorough evaluation of various influencing factors—including local urban and geographical characteristics, the composition of the farming population, and the rural economy—data collection was ultimately conducted in Xiaolou Town, Zengcheng District, Guangzhou City, Guangdong Province. This area is located in the suburbs of Guangzhou. According to a multi-year follow-up study by the Guangzhou Center for Disease Control and Prevention, residents of this district still retained a relatively complete traditional rural lifestyle (Lei et al., 2024). For example, these residents predominantly engaged in agricultural labor, walking, or cycling as their main modes of transportation and production, and their dietary structure relied heavily on locally produced agricultural products. Thus, this setting accurately reflected the living characteristics of rural residents.

**Figure 1:**
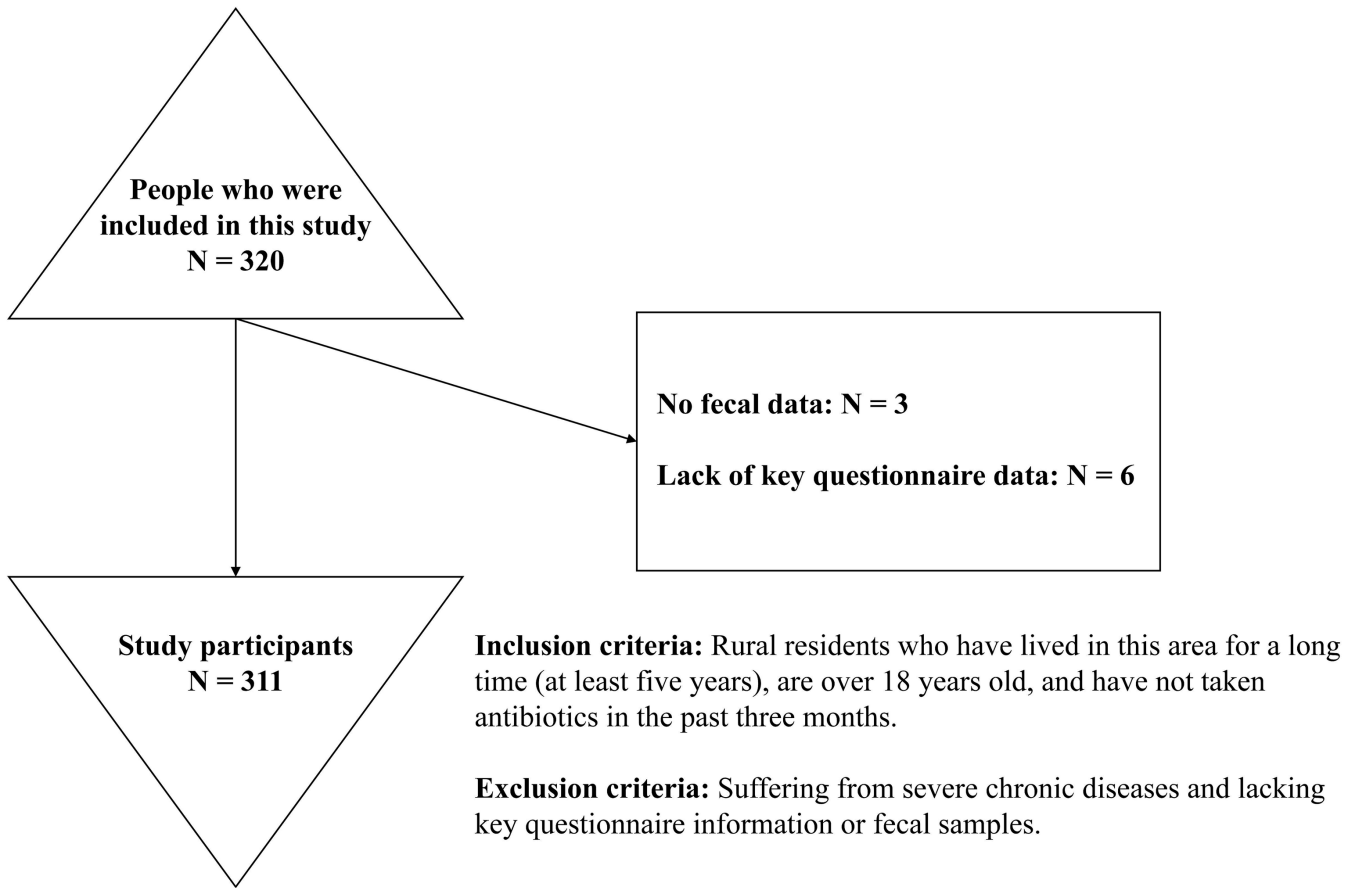
Flow diagram for the registration of participants.

Participants were recruited through community committee mobilization and telephone notification. The inclusion criteria were rural residents aged 18 years or older who had lived in this area for an extended period (at least 5 years) and had not taken antibiotics in the past 3 months. Exclusion criteria included rural residents with severe chronic diseases, as well as those lacking key questionnaire information or fecal samples. After obtaining informed consent, a professional team of 15 trained investigators, certified physicians, and nurses conducted questionnaire surveys, physical function tests, and fecal sample collection for all eligible participants. These staff members were proficient in local dialects such as Cantonese and Hakka to ensure smooth communication with local residents and accuracy of the questionnaire survey. This study was approved by the Ethics Committee of Guangzhou Center for Disease Control and Prevention (Approval No.: GZCDC-ECHR-2025P0061) and adhered to the principles of the Declaration of Helsinki. Written informed consent was obtained from all participants before enrollment. The study initially recruited 320 individuals, after which 9 participants with missing questionnaire data on lifestyle factors, fecal samples, or blood samples were excluded. Finally, 311 participants were included in subsequent analyses (Figure 1).

### 2.2 DNA extraction and 16S rRNA gene sequencing analysis

16S rRNA gene sequencing was performed by Wekemo Technology Co., Ltd. (Shenzhen, China). DNA was extracted following methods described in previous studies (Zheng et al., 2022). Genomic DNA was extracted from samples using the Cetyltrimethylammonium bromide (CTAB) method. DNA purity and concentration were assessed by 1% agarose gel electrophoresis. An appropriate amount of sample was placed in a centrifuge tube, and Milli-Q water was added to dilute the sample to a final concentration of 1 ng/μL. The V4 variable region was amplified by PCR using primers 515F (GTGCCAGCMGCCGCGGTAA) and 806R (GGACTACHVGGGTWTCTAAT). The amplification procedure was as follows: (1) Template: diluted genomic DNA; (2) Primers: specific primers with barcodes according to the selected sequencing region; (3) Enzyme and buffer: Phusion® High-Fidelity PCR Master Mix with GC Buffer (New England Biolabs). A high-efficiency and high-fidelity enzyme was used to ensure amplification efficiency and accuracy; (4) PCR reaction system and program (30 μL): Phusion Master Mix (2 ×)_15 μL, Forward Primer (1 μM/μL)_1 μL (1 μM), Reverse Primer (1 μM/μL)_1 μL (1 μM), gDNA (1 ng/μL)_10 μL (10 ng), H₂O_2 μL. Reaction program: initial denaturation at 98 °C for 1 min; 30 cycles of (98 °C for 10 s, 50 °C for 30 s, 72 °C for 30 s); final extension at 72 °C for 5 min; (5) PCR instrument: Bio-rad T100 gradient PCR thermocycler. PCR products were mixed in equal amounts based on concentration, then purified by electrophoresis on a 1 × TAE 2% agarose gel. The target bands were excised and recovered using a Universal DNA Purification Recovery Kit. Libraries were constructed using the NEB Next® Ultra DNA Library Prep Kit, then quality-checked and quantified using the Agilent 5400 system and q-PCR. After passing quality control, libraries were sequenced on the Illumina platform. Data were analyzed using the qiime tools import plugin to convert raw sequencing FASTQ files into a format compatible with QIIME2 and import them. Quality control, trimming, denoising, merging, and chimera removal were performed using the QIIME2 dada2 plugin to generate a feature table. A total of 31,187,730 high-quality sequences were obtained, with an average of 112,222 reads per sample (range: 50,587–168,690). For rarefaction, the data were rarefied to the minimum sequence count across all samples. The SILVA 138.2 database was trimmed to the V4 region according to the 515F/806R primer pair. The representative sequences of ASVs were then aligned against the trimmed SILVA 138.2 database using the QIIME2 feature-classifier plugin to generate a taxonomic classification table. Potentially contaminating mitochondrial and chloroplast sequences were filtered out using the QIIME2 feature-table plugin. Differential bacterial taxa between groups and among samples were identified using methods including ANCOM, ANOVA, Kruskal_Wallis test, LEfSe, and DESeq2. Diversity matrices were calculated using the QIIME2 core-diversity plugin. Alpha diversity indices (observed OTUs, Chao1, Shannon index, Simpson index, and Faith’s phylogenetic diversity index) were used to assess within-sample diversity. Beta diversity (Bray_Curtis index) was used to evaluate differences in microbial community structure between samples and was visualized using principal coordinate analysis (PCoA) plots.

### 2.3 Physical activity level and physical function assessment

Physical activity level was assessed using questions designed based on the International Physical Activity Questionnaire (IPAQ) short form, measuring the number of days and duration per day of different intensities of physical activity in the past 7 days across the domains of work, housework, transportation, and leisure. Given the limited research currently available on rural residents and the absence of a fully validated, standardized questionnaire specifically designed to quantify physical activity levels in agricultural labor, the IPAQ short form was adopted. As a World Health Organization-recommended and internationally recognized tool for physical activity assessment (Craig et al., 2003), the IPAQ has been validated for reliability and validity in multiple countries, including China, and thus represented the best available option for this study.

The intensity of physical activity was collected using the Rating of Perceived Exertion scale (Borg, 1982; MacIntosh et al., 2021). Finally, according to the IPAQ analysis guidelines (Craig et al., 2003), participants were classified into three physical activity levels: “high,” “moderate,” and “low.” PA (MET-minutes/week) = (days of vigorous activity × minutes of vigorous activity × 8.0) + (days of moderate activity × minutes of moderate activity × 4.0) + (days of walking × minutes of walking × 3.3) (Rosenberg et al., 2008).

Physical function parameters, including grip strength, back muscle strength, single-leg stance, and reaction time, were measured. Grip strength was assessed using an HHTC200WL grip strength tester (Guangzhou Huaxia Huihai Technology Co., Ltd., Guangzhou, China). Back muscle strength was measured with a BCS-400 electronic back dynamometer (Nantong Yuejian Physical Testing Equipment Co., Ltd., Nantong, China). Single-leg stance was evaluated using an HHTC200-BY closed-eye single-leg stance tester (Guangzhou Huaxia Huihai Technology Co., Ltd., Guangzhou, China). Reaction time was determined using an FYS-I electronic reaction time tester (Shanghai Kangfu Medical Instruments Co., Ltd., Shanghai, China). Each test was performed three times, and the mean values were used for subsequent analyses. Due to advanced age, limited mobility resulting from poor physical condition, or missing data, some participants did not undergo the physical function assessments. Ultimately, 265 participants were included in the analysis of physical function.

### 2.4 Covariates

Information on demographics, lifestyle, and disease status was collected through face-to-face interviews. Demographic characteristics included age, sex, education level, and household income. Additionally, lifestyle factors such as smoking and drinking status, physical activity, and diet were considered. Specifically, education level was categorized into four groups: no schooling, primary school, junior high school, and senior high school or above. Annual household income was divided into four categories: <4,000, 4,000–8,000, 8,000–12,000, and >12,000 CNY/year. Lifestyle questions inquired about sleep, smoking, and drinking habits among the rural population. Sleep status was assessed using questions such as “How has your sleep been in the past month?”, “Falling asleep quickly”, “Falling asleep slowly”, “Difficulty falling asleep”, and “Frequent insomnia.” A smoker was defined as someone who smoked at least one cigarette per day over the past 6 months. Similarly, a drinker was defined as a participant who consumed alcohol at least once per week over the past 6 months. Pesticide exposure was determined by asking whether the household currently stored pesticides. Conversely, former smokers/drinkers were defined as individuals who had quit smoking/drinking for more than 6 months. Physical fitness tests included height, weight, waist circumference, hip circumference, blood pressure, heart rate, dominant hand grip strength, back muscle strength, single-leg stance with eyes closed, choice reaction time, and body fat percentage. Body mass index (BMI) was calculated as weight in kilograms divided by height in meters squared. Hypertension was defined as systolic blood pressure (SBP) ≥ 140 mmHg or diastolic blood pressure (DBP) ≥ 90 mmHg. Disease status asked about conditions in the circulatory system (e.g., hypertension, coronary heart disease), endocrine system (e.g., hyperglycemia, diabetes, gout), digestive system (e.g., gastritis, gastric ulcer, hepatitis B), urinary system (e.g., nephritis, kidney stones), as well as tumors and thyroid diseases. Participants with any of the above conditions also reported their medication use over the past 3 months. Prior to statistical analysis, the following variables were coded as specified: (1) For age, participants aged <20 years were coded as 1; ≥20 and <30 years as 2; ≥30 and <40 years as 3; ≥40 and <50 years as 4; ≥50 and <60 years as 5; ≥60 and <70 years as 6; and ≥70 years as 7. (2) For weekly total physical activity level, low, moderate, and high levels were coded as 1, 2, and 3, respectively. (3) For sex, females were coded as 1 and males as 2. (4) For BMI, participants with BMI < 18.5 were coded as 1; ≥18.5 and <22.9 as 2; ≥23.0 and <27.5 as 3; and ≥27.5 as 4. (5) For exercise habits, those with regular exercise habits were coded as 1 and those without as 2. (6) For sleep quality over the preceding 3 months, participants who fell asleep quickly were coded as 1; those with normal sleep onset as 2; those with difficulty falling asleep as 3; and those with frequent insomnia as 4. (7) For smoking status, non-smokers were coded as 1 and smokers as 2. (8) For alcohol consumption, participants with drinking habits were coded as 1 and those without as 2. (9) For pesticide exposure, those with pesticide exposure were coded as 1 and those without as 2.

### 2.5 Statistical analysis

In this study, standard descriptive statistics [counts, proportions, means (SD)] were used to describe participant characteristics. As the data did not follow a normal distribution, the Kruskal–Wallis test was used to compare differences in alpha diversity indices among rural residents with different physical activity levels, followed by *post hoc* pairwise comparisons using the stepwise descending method. Permutational multivariate analysis of variance (PERMANOVA) was used to analyze weighted UniFrac distances. A *p*-value < 0.05 indicated statistical significance. Statistical analyses were performed using SPSS version 27.0. PCoA of weighted UniFrac distances was used to assess microbiome variation among fecal samples in QIIME 2. Spearman’s rank correlation coefficient was calculated to evaluate associations between gut microbiota and physical activity level, BMI, age, drinking status, smoking status, pesticide exposure, sleep quality, and exercise status. Subsequently, multiple regression analysis was performed to calculate the regression coefficient B and *p-*value for differentially abundant genera, the variance inflation factor (VIF) for all independent variables was less than 5, indicating that the regression results were stable. This study was an exploratory cross-sectional analysis in which multiple statistical tests were performed. Given the exploratory nature of this study and to avoid missing potentially weak signals due to overly conservative corrections, we did not apply any form of correction for multiple comparisons. Accordingly, all reported *p*-values are nominal *p*-values and have not been adjusted for multiple testing. Caution should be exercised when interpreting these results; a *p*-value < 0.05 indicates statistical significance only at the single-test level and does not imply significance after correction for multiple comparisons. Confirmatory studies in independent cohorts are needed. To test for potential nonlinear associations between physical activity level and gut microbiota, hierarchical regression analysis was used.

## 3 Results

### 3.1 Characteristics of the study population

In this study, a total of 311 participants were recruited and analyzed. The characteristics and health status of the participants are shown in Table 1. The mean (SD) age and BMI were 57.04 (14.22) years and 23.66 (3.59) kg/m^2^, respectively. The educational level of participants was mainly primary school (42.8%) and junior high school (31.5%). The majority of participants were non-smokers (68.5%) and non-drinkers (85.5%). The annual household income distribution was as follows: 17.4% below 4,000 CNY, 29.3% between 4,000 and 8,000 CNY, 14.8% between 8,000 and 12,000 CNY, and 38.6% above 12,000 CNY. Regarding sleep status, most participants reported normal or rapid sleep onset, while a small proportion (18%) experienced frequent difficulty falling asleep or insomnia. The vast majority of participants did not store pesticides at home (92.8%). Most participants engaged in high levels of physical activity (66.9%), with a minority engaging in low levels (9.97%). Among all participants, the majority participated in agricultural labor or housework (85.53%), while only 14.47% did not engage in such activities. Additionally, half of the participants had a daily exercise habit.

**Table 1:** Characteristics of the study participants and their physical activity levels (*N* = 311).

Classification	Overall (*N* = 311)	Low-level physical activity (*N* = 31)	Moderate-level physical activity (*N* = 72)	High-level physical activity (*N* = 208)	*p*-value
Age, year, mean (SD)	57.04 (14.22)	54.81 (16.16)	53.86 (17.66)	58.48 (12.32)	0.106
BMI, kg/m^2^, mean (SD)	23.66 (3.59)	23.84 (5.05)	23.13 (3.67)	23.82 (3.29)	0.297
Sex, *n* (%)					<0.001
Male	172 (55.30)	29 (93.50)	52 (72.20)	91 (43.80)	
Female	139 (44.70)	2 (6.50)	20 (27.80)	117 (56.30)	
Education, *n* (%)					0.035
Illiteracy	18 (5.80)	1 (3.20)	4 (5.60)	13 (6.30)	
Primary school	133 (42.8)	9 (29.0)	26 (36.1)	98 (47.1)	
Junior high school	98 (31.5)	10 (35.5)	20 (27.8)	67 (32.2)	
High school or above	62 (19.9)	10 (32.3)	22 (30.6)	30 (14.4)	
Household income, 1,000 CNY, *n* (%)					0.308
<4	54 (17.40)	5 (16.10)	10 (13.90)	39 (18.80)	
4–8	91 (29.30)	9 (29.00)	15 (20.80)	67 (32.20)	
8–12	46 (14.80)	3 (9.70)	12 (16.70)	31 (14.90)	
>12	120 (38.60)	14 (45.20)	35 (48.60)	71 (34.10)	
Type of labor, *n* (%)					<0.001
No work	45 (14.47)	26 (8.36)	17 (23.61)	2 (0.96)	
Agricultural labor	18 (5.79)	5 (16.13)	6 (8.33)	7 (3.37)	
Household chores	164 (52.73)	0	42 (58.33)	122 (58.65)	
Both of them do	84 (27.01)	0	7 (9.72)	77 (37.1)	
Exercise fatigue level, *n* (%)					<0.001
Yes	156 (50.20)	30 (96.80)	38 (52.80)	88 (42.30)	
No	155 (49.80)	1 (3.20)	34 (47.20)	120 (57.70)	
Sleep quality, *n* (%)					0.302
Very good	150 (48.20)	18 (58.10)	27 (37.50)	105 (50.50)	
Ordinary	105 (33.80)	10 (32.30)	28 (38.90)	67 (32.20)	
Worse	50 (16.10)	2 (6.50)	16 (22.20)	32 (15.40)	
Very bad	6 (1.90)	1 (3.20)	1 (1.40)	4 (1.90)	
Smoking status, *n* (%)					0.002
Yes	98 (31.5)	18 (58.10)	25 (34.70)	55 (26.40)	
No	213 (68.5)	13 (41.90)	47 (65.3)	153 (73.6)	
Drinking status, *n* (%)					0.368
Yes	45 (14.50)	6 (19.40)	13 (18.10)	26 (12.50)	
No	266 (85.50)	25 (80.6)	59 (81.9)	182 (87.5)	
Pesticide exposure, *n* (%)					0.299
Yes	22 (7.10)	1 (3.20)	3 (4.20)	18 (8.70)	
No	289 (92.80)	30 (96.80)	69 (95.80)	190 (91.30)	
MET (SD)					< 0.001
	3853.97 (2311.83)	401.03 (1091.08)	1850.17 (791.62)	5062.22 (1722.61)	

BMI, Body mass index; CNY, Chinese yuan; SD, Standard deviation; MET: Metabolic Equivalent of Task.

### 3.2 Alpha diversity of gut microbiota in rural residents with different physical activity levels

**Figure 2:**
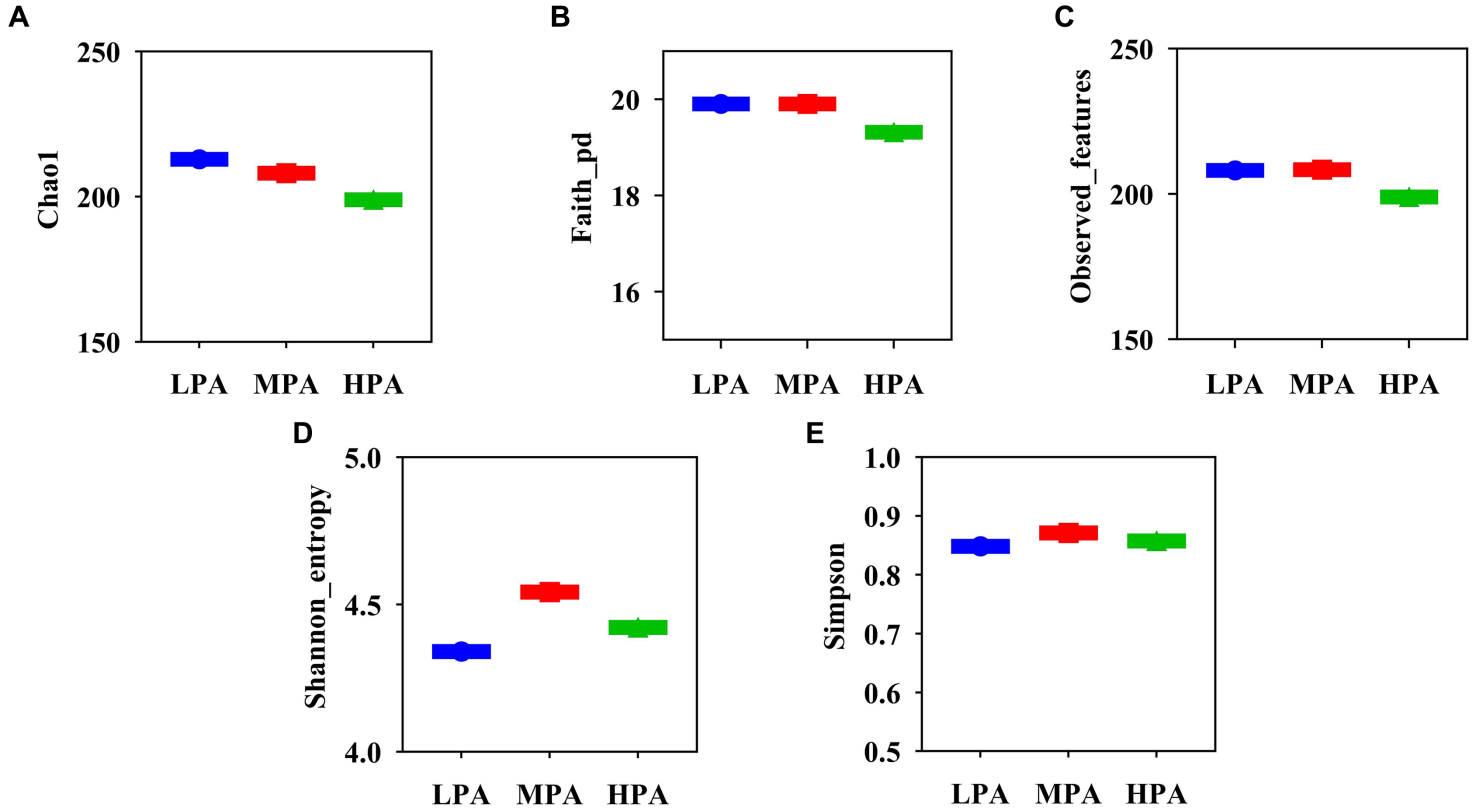
Alpha diversity index of farmers with different levels of physical activity. **(A)** Chao1, **(B)** Faith_pd, **(C)** Observed_features, **(D)** Shannon_entropy index, **(E)** Simpson index. LPA (Low physical activity, *N* = 31), MPA (Moderate physical activity, *N* = 72), HPA (High physical activity, *N* = 208).

Figure 2 shows the alpha diversity indices of rural residents with different physical activity levels. Alpha diversity reflects the species diversity of samples, including the richness and evenness of microbial composition. The observed_features index (Figure 2C) refers to the actual number of OTUs detected in a sample and is an indicator of OTU richness. Faith’s phylogenetic diversity index (faith_pd) (Figure 2B) is a diversity index calculated based on a phylogenetic tree. A higher faith_pd value indicates greater evolutionary divergence and higher diversity. The Chao1 index (Figure 2A) is an indicator of species richness, estimating theoretical richness from observed data and approximating true richness. The Shannon index (Figure 2D) increases with community richness and evenness. In contrast, the Simpson index was a similarity index (higher values indicated lower diversity). The Simpson index was calculated as *D* = *Σ pᵢ*^2^, where *pᵢ* represented the relative abundance of the i-th genus (Figure 2E). It reflected the proportional abundance of species in a sample.

The results showed that both the moderate physical activity (MPA) group and the high physical activity (HPA) group had higher Shannon indices than the low physical activity (LPA) group, with the MPA group exhibiting a higher Shannon index than the HPA group. The Simpson index confirmed this trend; although the LPA group had a lower Simpson index, suggesting more even distribution among species, the overall differences among the three groups were small. In contrast, the Chao1 index showed that the overall position of the boxplot for the LPA group was higher than those of the MPA and HPA groups. The faith_pd index showed similar boxplot positions for the LPA and MPA groups, both higher than that of the HPA group. Notably, the distribution trend of the observed_features index across groups was similar to that of the Chao1 index, with the LPA group’s boxplot positioned higher than that of the MPA group, and the MPA group higher than the HPA group. Using the Kruskal–Wallis test, there were no significant differences among the groups for the Chao1, Shannon, Simpson, faith_pd, or observed_features indices.

### 3.3 Taxonomic composition analysis of gut microbiota in rural residents with different physical activity levels

**Figure 3:**
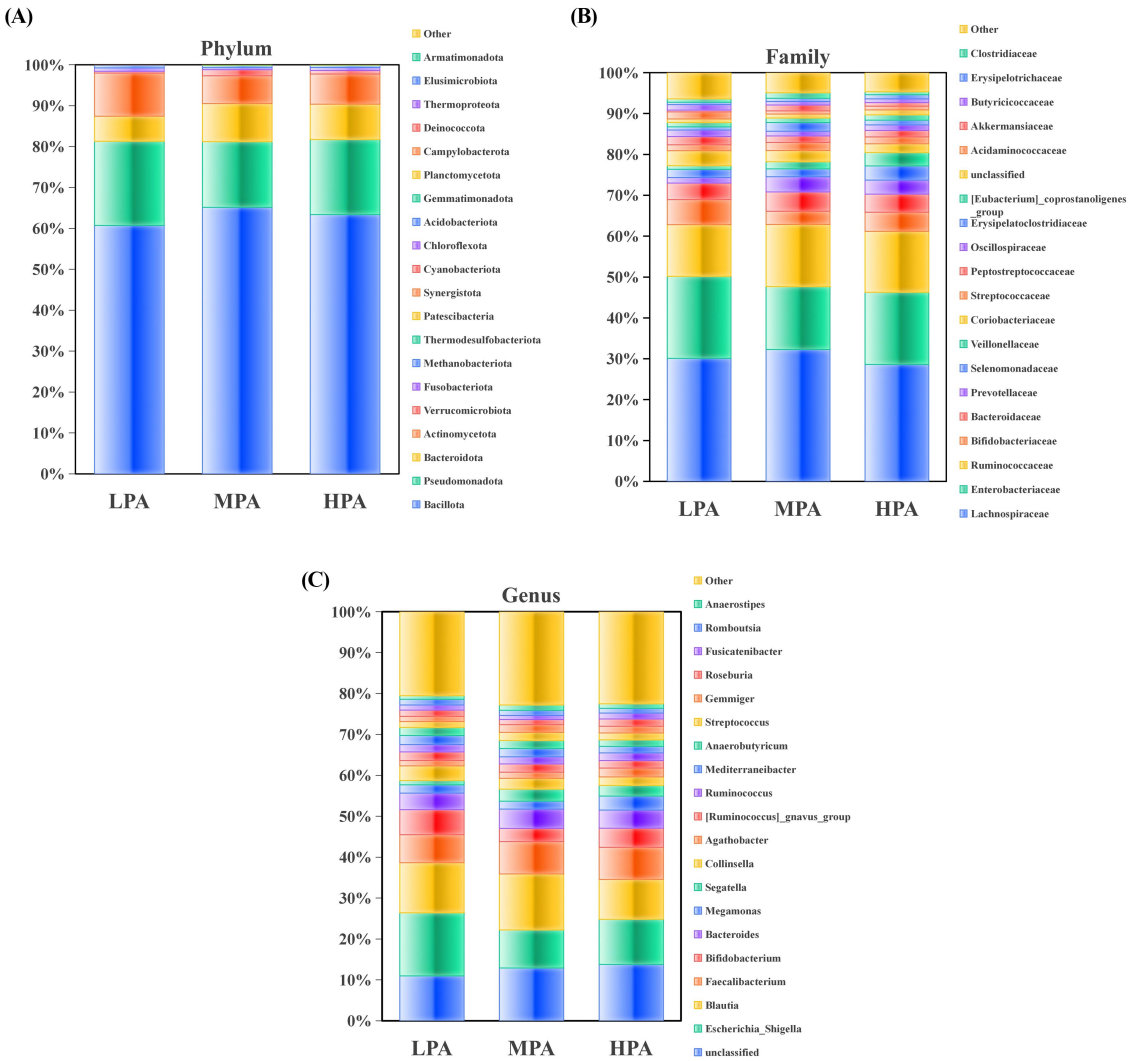
Relative abundance of bacterial taxa in populations with different physical activity levels. **(A)** Relative abundance of bacteria at the phylum level in populations with different physical activity levels. **(B)** Relative abundance of bacteria at the family level in populations with different physical activity levels. **(C)** Relative abundance of bacteria at the genus level in populations with different physical activity levels. LPA (Low physical activity, *N* = 31), MPA (Moderate physical activity, *N* = 72), HPA (High physical activity, *N* = 208).

As shown in Figure 3, species composition analysis revealed that at the phylum level (Figure 3A), the microbial community structures of the HPA, MPA, and LPA groups were all dominated by Bacillota, Pseudomonadota, Bacteroidota, and Actinomycetota, with their combined relative abundance exceeding 98% in each group. Other low-abundance phyla included Verrucomicrobiota, Fusobacteriota, and Methanobacteriota. Differences in the relative abundances of major phyla were observed among the three groups. Bacillota was highest in the MPA group (65.16%), followed by the HPA group (63.39%), and lowest in the LPA group (60.74%). Pseudomonadota was highest in the LPA group (20.54%), intermediate in the HPA group (18.37%), and lowest in the MPA group (16.05%). Bacteroidota was also highest in the MPA group (9.31%), followed by the HPA group (8.59%), and lowest in the LPA group (6.14%). Actinomycetota was significantly higher in the LPA group than in the other two groups (LPA 10.58% vs. HPA 7.37% vs. MPA 6.78%). Verrucomicrobiota was highest in the MPA group (1.47%), followed by the HPA group (0.88%), and lowest in the LPA group (0.42%). Fusobacteriota and Methanobacteriota showed higher trends in the LPA group (0.82 and 0.65%, respectively).

At the family level (Figure 3B), Lachnospiraceae was highest in the MPA group (32.26%), followed by the LPA group (30.11%), and lowest in the HPA group (28.61%). Enterobacteriaceae was highest in the LPA group (20.06%), intermediate in the HPA group (17.67%), and lowest in the MPA group (15.40%). Ruminococcaceae was similar in the MPA and HPA groups (15.18 and 14.90%, respectively), both higher than in the LPA group (12.64%). Bifidobacteriaceae was significantly higher in the LPA group than in the other two groups (LPA 6.16% vs. HPA 4.67% vs. MPA 3.25%). Bacteroidaceae was highest in the MPA group (4.73%), followed by the HPA group (4.43%), and lowest in the LPA group (4.01%). Prevotellaceae was also highest in the MPA group (3.70%), followed by the HPA group (3.45%), and lowest in the LPA group (1.39%). Akkermansiaceae was highest in the MPA group (1.47%), followed by the HPA group (0.88%), and lowest in the LPA group (0.42%). Veillonellaceae was highest in the HPA group (3.27%), followed by the MPA group (1.78%), and lowest in the LPA group (0.84%). Acidaminococcaceae was highest in the LPA group (1.75%), significantly higher than in the HPA group (0.90%) and MPA group (0.78%). Butyricicoccaceae was also highest in the LPA group (1.34%), compared to 0.89 and 0.87% in the HPA and MPA groups, respectively. Other families such as Coriobacteriaceae, Streptococcaceae, Peptostreptococcaceae, Oscillospiraceae, Erysipelotrichaceae, and Clostridiaceae also showed minor differences among groups.

At the genus level (Figure 3C), *Escherichia_Shigella* was highest in the LPA group (15.38%), followed by the HPA group (11.03%), and lowest in the MPA group (9.29%). *Blautia* was highest in the MPA group (13.70%), followed by the LPA group (12.28%), and lowest in the HPA group (9.74%). Similarly, *Faecalibacterium* was similar in the MPA and HPA groups (7.89 and 7.87%, respectively), both higher than in the LPA group (6.81%). Additionally, *Anaerostipes* was also highest in the MPA group (1.32%), followed by the HPA group (1.14%), and lowest in the LPA group (0.83%). Notably, the significant increase in *Bifidobacterium* in the LPA group contrasted with the decrease in other beneficial genera. *Bifidobacterium* was highest in the LPA group (6.16%), followed by the HPA group (4.67%), and lowest in the MPA group (3.25%). Furthermore, *Segatella* was highest in the MPA group (2.93%), followed by the HPA group (2.61%), and lowest in the LPA group (1.09%). In contrast, *Roseburia* was highest in the HPA group (1.77%), followed by the LPA group (1.57%), and lowest in the MPA group (1.24%). The abundance of *Mediterraneibacter* was highest in the LPA group (2.22%), followed by the MPA group (2.00%), and lowest in the HPA group (1.57%).

### 3.4 Beta diversity of gut microbiota in rural residents with different physical activity levels

**Figure 4:**
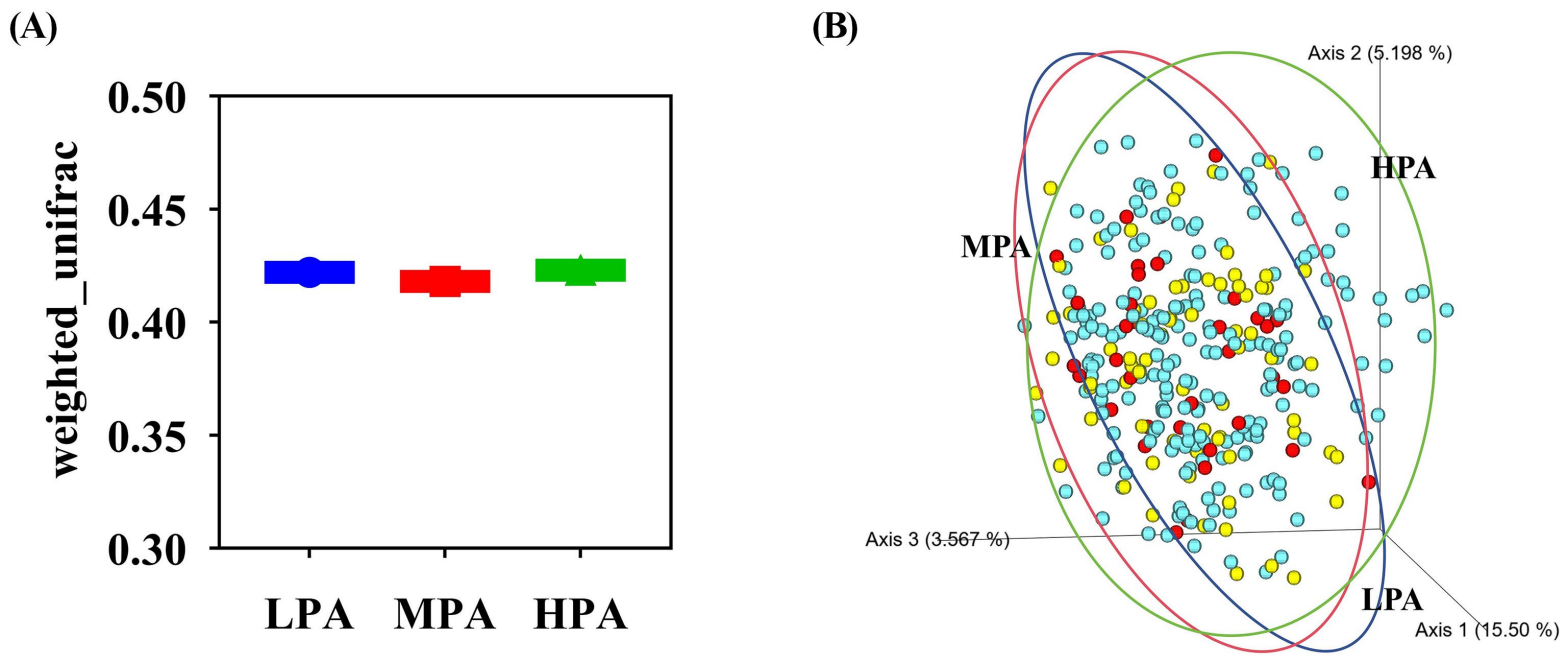
Beta diversity. **(A)** Statistical plot of beta diversity significance among populations with different physical activity levels. *R*^2^ = 0.006, *p* = 0.589. **(B)** Principal coordinate analysis (PCoA) plot of populations with different physical activity levels. The PCoA plot visualizes weighted UniFrac distances. The X-axis represents the first principal component (Axis 1), and the percentage indicates the proportion of data variance explained by Axis 1. The Y-axis represents the second principal component (Axis 2), and the percentage indicates the proportion of variance explained by Axis 2. Each point in the PCoA plot represents one sample, and samples from the same group are shown in the same color. The PCoA plot shows the distribution of samples along the principal components based on weighted UniFrac distances. LPA (Low physical activity, *N* = 31), MPA (Moderate physical activity, *N* = 72), HPA (High physical activity, *N* = 208).

As shown in Figure 4 based on weighted UniFrac distance PCoA (Figure 4B), the elliptical areas of the three groups almost completely overlapped, with no clear separation trend observed. Axis 1 explained only 15.5% of the variation, Axis 2 explained 5.198%, and Axis 3 explained 3.567%. However, the Permutational multivariate analysis of variance (PERMANOVA) showed no significant differences among the LPA, MPA, and HPA groups (*p* > 0.05). Although differences in the relative abundances of multiple phyla, families, and genera were observed among the different physical activity groups (Figure 4A), beta diversity analysis did not detect significant separation in community structure.

### 3.5 LEfSe analysis of gut microbiota in rural residents with different physical activity levels

**Figure 5:**
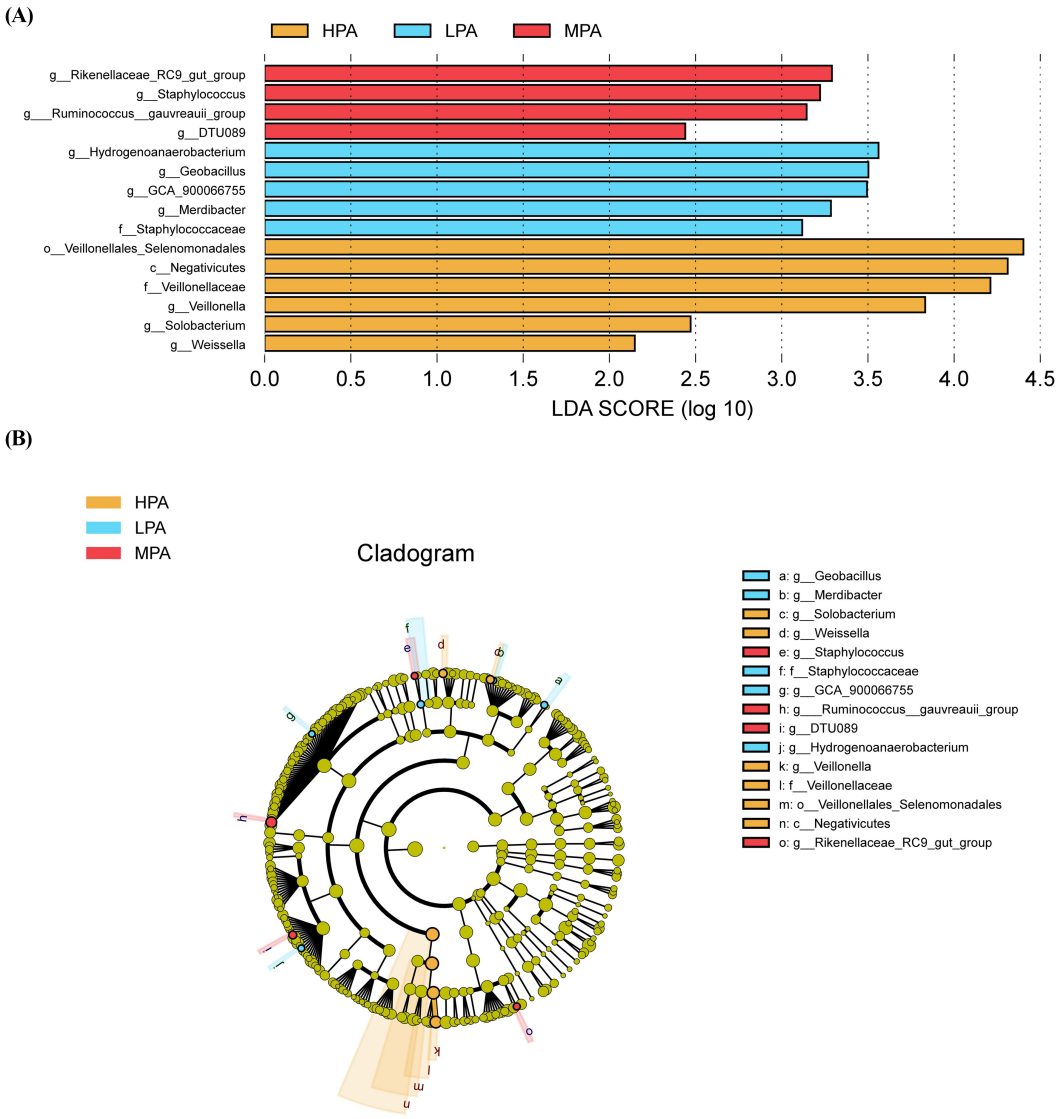
LEfSe analysis. **(A)** LDA scores of different taxonomic levels [the low physical activity (LPA, *N* = 31), the moderate physical activity (MPA, *N* = 72), the high physical activity (HPA, *N* = 208)]. The figure shows differences in abundance of various genera or families. Higher LDA scores indicate greater importance of the corresponding taxon in the respective group. **(B)** Cladogram showing abundance differences among different taxa (LPA, MPA, HPA). Color-coded regions represent group-specific abundances, nodes represent different taxonomic levels, and line thickness indicates relative abundance magnitude.

As shown in Figure 5, different microbial taxa showed (Figure 5A) enrichment trends across the physical activity groups (LDA > 2). In the HPA group, *o_Veillonellales_Selenomonadales*, *c_Negativicutes*, *f_Veillonellaceae*, *g_Veillonella*, *g_Solobacterium*, and *g_Weissella* were significantly enriched. In the LPA group, *g_Hydrogenoanaerobacterium*, *g_Geobacillus*, *g_GCA 900066755*, *g_Merdibacter*, and *f_Staphylococcaceae* showed higher abundance. In the MPA group, *g_Rikenellaceae_RC9_gut_group*, *g_Staphylococcus*, *g_Ruminococcus_gauvreauii_group*, and *g_DTU089* were significantly enriched.

### 3.6 Correlation between covariates and abundance of specific gut microbial genera

Table 2 presents the correlations between various covariates and the abundance of specific gut microbial genera. The results showed that only a few genera exhibited statistically significant correlations with physical activity level. *Segatella* (*r* = 0.113, *p* = 0.047) increased with higher physical activity levels, showing a significant positive correlation. *Blautia* (*r* = −0.143, *p* = 0.012) and *[Ruminococcus]_gnavus_group* (*r* = −0.127, *p* = 0.025) decreased with increasing physical activity levels, showing significant negative correlations.

**Table 2:** Correlation coefficient between gut microbiota and covariates.

Genus	Physical activity level		Sex		Age		BMI		Exercise	
	*r*	*p*-value	*r*	*p*-value	*r*	*p*-value	*r*	*p*-value	*r*	*p*-value
*Unclassified*	0.031	0.587	−0.040	0.487	0.234	<0.001	0.008	0.882	−0.030	0.595
*Escherichia_Shigella*	0.032	0.576	−0.017	0.766	0.227	<0.001	0.192	<0.001	0.015	0.798
*Blautia*	−0.143	0.012	−0.042	0.459	−0.288	<0.001	0.014	0.806	0.012	0.835
*Faecalibacterium*	0.064	0.258	0.004	0.938	−0.146	0.010	0.047	0.405	−0.020	0.719
*Bifidobacterium*	−0.014	0.811	0.178	0.002	−0.210	<0.001	0.002	0.965	−0.035	0.542
*Bacteroides*	−0.046	0.416	0.110	0.052	0.046	0.417	−0.121	0.033	0.024	0.679
*Megamonas*	0.036	0.530	0.011	0.852	−0.120	0.035	0.070	0.217	−0.022	0.700
*Segatella*	0.113	0.047	−0.073	0.199	0.045	0.430	−0.027	0.641	0.047	0.408
*Collinsella*	−0.096	0.090	−0.233	<0.001	−0.090	0.112	0.026	0.653	−0.011	0.848
*Agathobacter*	0.074	0.191	0.082	0.147	−0.007	0.906	0.053	0.353	0.113	0.046
*[Ruminococcus]_gnavus_group*	−0.127	0.025	−0.038	0.507	−0.114	0.045	−0.043	0.455	−0.033	0.559
*Ruminococcus*	0.047	0.407	0.005	0.931	0.157	0.006	0.0004	0.995	0.001	0.981
*Mediterraneibacter*	−0.036	0.529	−0.104	0.067	−0.161	0.004	−0.010	0.864	−0.056	0.325
*Anaerobutyricum*	−0.049	0.385	−0.111	0.050	−0.085	0.134	0.018	0.752	−0.043	0.449
*Streptococcus*	−0.012	0.829	0.007	0.903	−0.099	0.081	0.001	0.980	−0.081	0.156
*Gemmiger*	0.044	0.441	−0.064	0.259	−0.071	0.209	−0.040	0.679	0.037	0.510
*Roseburia*	0.039	0.493	−0.048	0.394	0.122	0.032	−0.020	0.728	0.034	0.552
*Fusicatenibacter*	0.039	0.494	0.115	0.043	−0.283	<0.001	0.016	0.782	−0.045	0.427
*Romboutsia*	−0.066	0.244	−0.142	0.012	0.107	0.059	−0.026	0.648	0.075	0.187
*Anaerostipes*	0.009	0.871	0.063	0.269	−0.158	0.005	−0.103	0.069	−0.055	0.337

Genus	Pesticide exposure		Sleep quality		Smoking status		Drinking Status	
	*r*	*p*-value	*r*	*p*-value	*r*	*p*-value	*r*	*p*-value
*Unclassified*	−0.109	0.054	−0.019	0.745	0.004	0.938	−0.037	0.521
*Escherichia_Shigella*	0.006	0.918	0.024	0.673	−0.066	0.245	−0.074	0.192
*Blautia*	0.062	0.274	−0.019	0.735	0.101	0.075	0.123	0.031
*Faecalibacterium*	−0.026	0.649	−0.113	0.047	0.034	0.554	0.030	0.595
*Bifidobacterium*	0.050	0.381	−0.012	0.828	−0.164	0.004	−0.039	0.492
*Bacteroides*	0.017	0.763	0.006	0.914	−0.061	0.284	−0.021	0.711
*Megamonas*	−0.049	0.392	0.013	0.824	−0.063	0.268	0.067	0.237
*Segatella*	−0.055	0.336	−0.044	0.440	0.100	0.079	−0.111	0.050
*Collinsella*	−0.001	0.979	−0.051	0.374	0.146	0.010	0.126	0.026
*Agathobacter*	−0.088	0.121	−0.116	0.040	−0.011	0.844	−0.022	0.694
*[Ruminococcus]_gnavus_group*	0.017	0.765	0.058	0.310	0.110	0.054	0.131	0.021
*Ruminococcus*	−0.093	0.103	−0.057	0.318	0.017	0.764	−0.074	0.192
*Mediterraneibacter*	−0.036	0.525	−0.056	0.328	0.054	0.339	0.108	0.057
*Anaerobutyricum*	−0.106	0.062	−0.113	0.047	0.096	0.092	0.065	0.253
*Streptococcus*	0.086	0.128	0.103	0.071	0.034	0.554	−0.104	0.066
*Gemmiger*	−0.070	0.219	−0.118	0.037	−0.020	0.730	0.0001	0.996
*Roseburia*	−0.123	0.031	−0.105	0.065	0.086	0.128	0.003	0.963
*Fusicatenibacter*	0.077	0.176	−0.162	0.004	−0.044	0.439	−0.022	0.699
*Romboutsia*	−0.289	<0.001	−0.027	0.640	0.110	0.052	0.029	0.605
*Anaerostipes*	−0.039	0.497	−0.025	0.656	−0.014	0.806	0.010	0.862

BMI, Body mass index.

Notably, age was the variable correlated with the greatest number of genera. The relative abundances of unclassified genus (*r* = 0.234, *p* < 0.001), *Escherichia_Shigella* (*r* = 0.227, *p* < 0.001), *Ruminococcus* (*r* = 0.157, *p* = 0.006), and *Roseburia* (*r* = 0.122, *p* = 0.032) increased with age. In contrast, the abundances of *Blautia* (*r* = −0.288, *p* < 0.001), *Faecalibacterium* (*r* = −0.146, *p* = 0.01), *Bifidobacterium* (*r* = −0.210, *p* < 0.001), *Megamonas* (*r* = −0.120, *p* = 0.032), *[Ruminococcus]_gnavus_group* (*r* = −0.114, *p* = 0.045), *Mediterraneibacter* (*r* = −0.161, *p* = 0.004), *Fusicatenibacter* (*r* = −0.283, *p* < 0.001), and *Anaerostipes* (*r* = −0.158, *p* = 0.005) decreased with age. *Bifidobacterium* (*r* = 0.178, *p* = 0.002) and *Fusicatenibacter* (*r* = 0.115, *p* = 0.043) were more abundant in females. *Collinsella* (*r* = −0.233, *p* < 0.001), *Romboutsia* (*r* = −0.142, *p* = 0.012), and *Anaerobutyricum* (*r* = −0.111, *p* = 0.05) were more abundant in males. *Escherichia_Shigella* (*r* = 0.192, *p* < 0.001) was significantly positively correlated with BMI, while *Bacteroides* (*r* = −0.121, *p* = 0.033) and Other (*r* = −0.123, *p* = 0.031) were significantly negatively correlated with BMI. Pesticide exposure was associated with significantly increased abundances of *Romboutsia* (*r* = −0.289, *p* < 0.001) and *Roseburia* (*r* = −0.123, *p* = 0.031). *Faecalibacterium* (*r* = −0.113, *p* = 0.047), *Agathobacter* (*r* = −0.116, *p* = 0.040), *Gemmiger* (*r* = −0.118, *p* = 0.037), *Anaerobutyricum* (*r* = 0.113, *p* = 0.047), and *Fusicatenibacter* (*r* = 0.162, *p* = 0.004) were all significantly correlated with sleep quality (negative correlations for *Faecalibacterium*, *Agathobacter*, *Gemmiger*; positive for *Anaerobutyricum* and *Fusicatenibacter*). *Collinsella* (*r* = 0.146, *p* = 0.010) was significantly more abundant in smokers, while *Bifidobacterium* (*r* = −0.164, *p* = 0.004) was significantly less abundant in smokers. *Blautia* (*r* = 0.123, *p* = 0.031), *Collinsella* (*r* = 0.126, *p* = 0.026), and *[Ruminococcus]_gnavus_group* (*r* = 0.131, *p* = 0.021) were more abundant in drinkers.

### 3.7 Correlation between physical function and physical activity level

Table 3 presents the correlations between physical function and physical activity level. The results revealed that muscular strength and reaction speed were significantly associated with physical activity level in this population, whereas balance ability showed no significant association. Grip strength (*r* = −0.172, *p* = 0.005) and back muscle strength (*r* = −0.220, *p* < 0.001) were significantly negatively correlated with physical activity level; individuals with higher physical activity levels exhibited lower absolute grip strength and back muscle strength. In contrast, reaction time (*r* = 0.207, *p* < 0.001) was significantly positively correlated with physical activity level, indicating that higher physical activity levels were associated with longer reaction times (i.e., slower reaction speed). Left single-leg stance with eyes closed (seconds) (*r* = −0.057, *p* = 0.355) and right single-leg stance with eyes closed (seconds) (*r* = −0.114, *p* = 0.065) showed no significant correlation with physical activity level.

**Table 3:** Correlation coefficient between physical functions and physical activity level.

Variate	Physical activity level (*N* = 265)	
	*r*	*p*-value
The power of gripping	−0.172	0.005
Back muscle strength	−0.220	<0.001
Standing on one foot with the left eye closed	−0.057	0.355
Standing on one foot with the right eye closed	−0.114	0.065
Reaction time	0.207	<0.001

### 3.8 Multiple regression analysis

**Figure 6:**
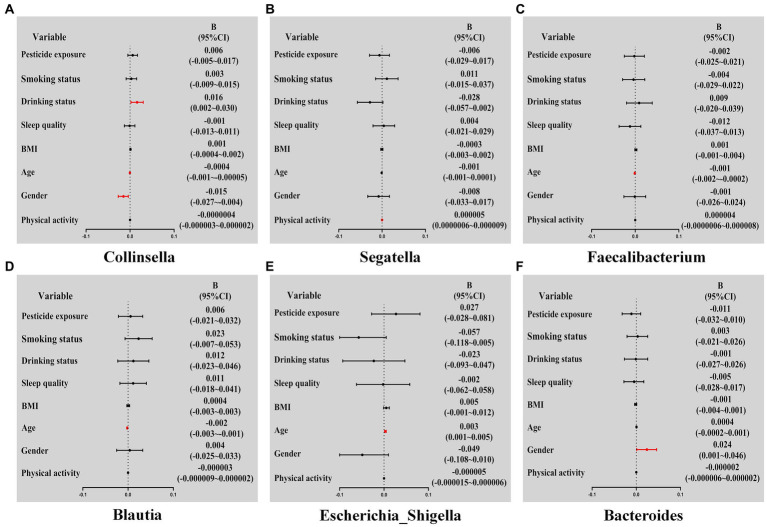
Univariate and multivariate regression analysis of gut microbiota. **(A)** *Collinsella*, **(B)** *Segatella*, **(C)** *Faecalibacterium*, **(D)** *Blautia*, **(E)** *Escherichia_Shigella*, **(F)** *Bacteroides*. Multivariate regression analysis adjusted for physical activity level, sex, age, BMI, sleep quality, drinking status, smoking status, and pesticide exposure. BMI, Body mass index.

**Figure 7:**
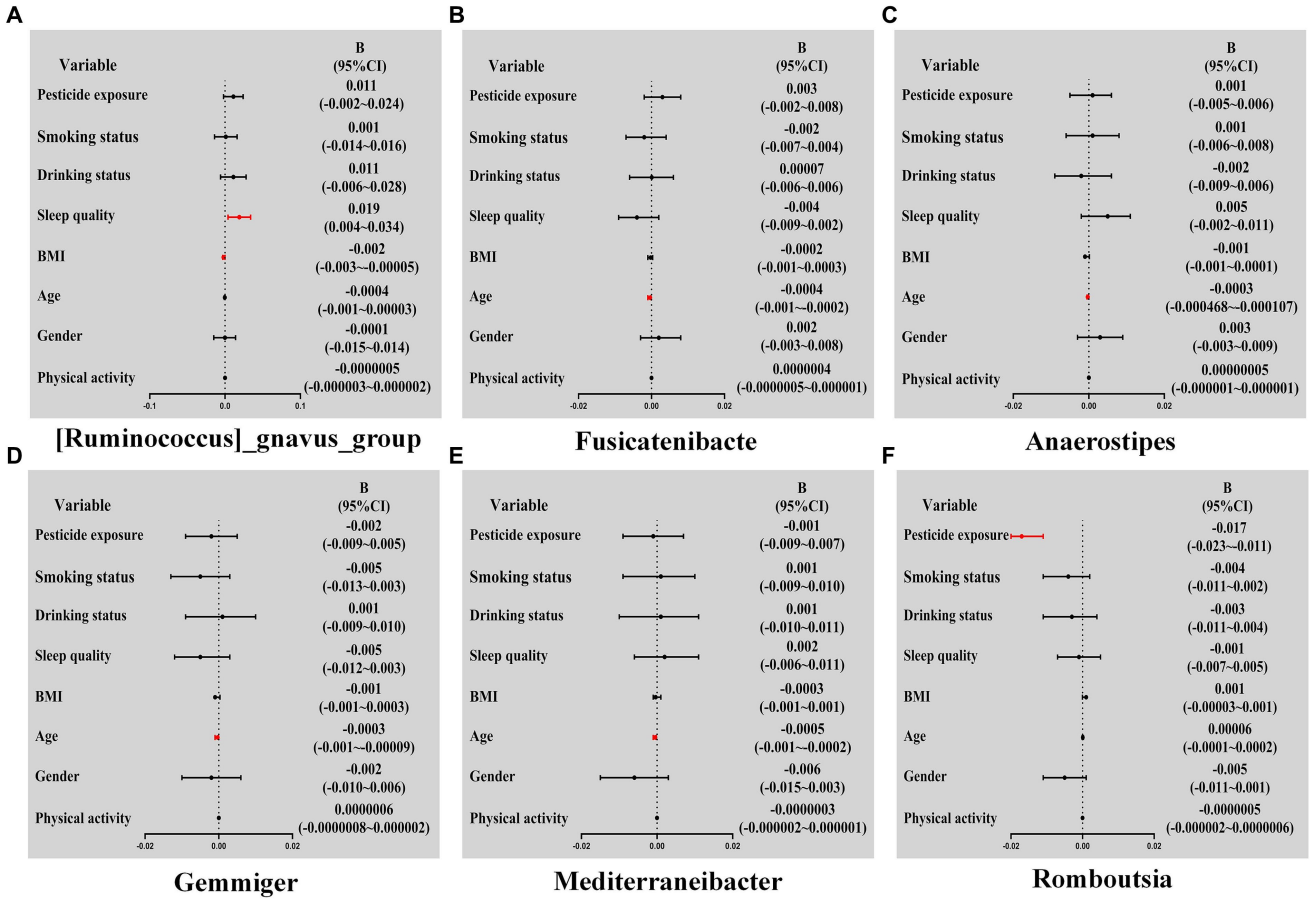
Univariate and multivariate regression analysis of gut microbiota. **(A)** *[Ruminococcus]_gnavus_group*, **(B)** *Fusicatenibacter*, **(C)** *Anaerostipes*, **(D)** *Gemmiger*, **(E)** *Mediterraneibacter*, **(F)** *Romboutsia*. Multivariate regression analysis adjusted for physical activity level, sex, age, BMI, sleep quality, drinking status, smoking status, and pesticide exposure. BMI, Body mass index.

**Figure 8:**
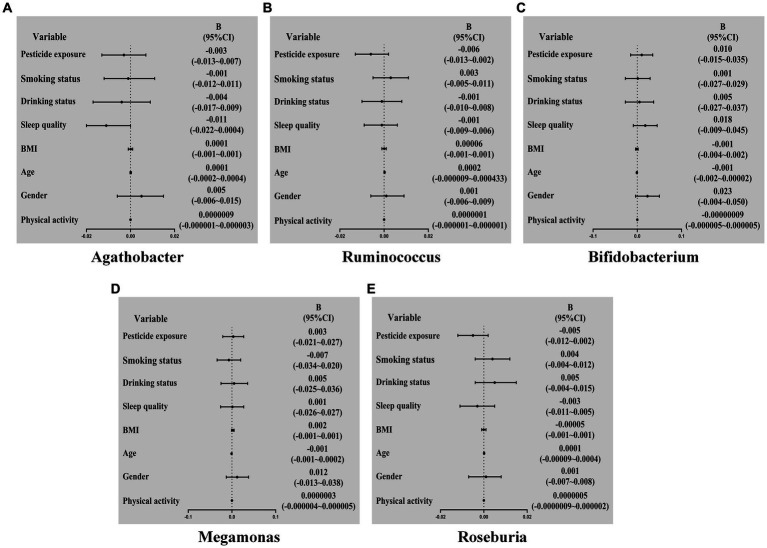
Univariate and multivariate regression analysis of gut microbiota. **(A)** *Agathobacter*, **(B)** *Ruminococcus*, **(C)** *Bifidobacterium*, **(D)** *Megamonas*, **(E)** *Roseburia*. Multivariate regression analysis adjusted for physical activity level, sex, age, BMI, sleep quality, drinking status, smoking status, and pesticide exposure. BMI, Body mass index.

Multiple regression analysis was performed to examine the associations between gut microbiota and physical activity level, sex, age, sleep quality, smoking and drinking status, and pesticide exposure. The results showed that physical activity level was significantly positively associated only with the genus *Segatella* [B (95% CI): 0.000005 (0.0000006, 0.0000009)] (Figure 6B), but not with any of the other genera analyzed (all *p* > 0.05). Sex was significantly associated with *Bacteroides* [B (95% CI): 0.024 (0.001, 0.046)] (Figure 6F) and *Collinsella* [B (95% CI): −0.015 (−0.027, −0.004)] (Figure 6A), indicating differences between sexes. Age was significantly associated with multiple genera: negatively associated with *Blautia* [B (95% CI): −0.002 (−0.003, −0.001)] (Figure 6D), *Anaerostipes* [B (95% CI): −0.0003 (−0.000468, −0.000107)] (Figure 7C), *Faecalibacterium* [B (95% CI): −0.001 (−0.026, 0.024)] (Figure 6C), *Mediterraneibacter* [B (95% CI): −0.0005 (−0.001, −0.0002)] (Figure 7E), *Collinsella* [B (95% CI): −0.0004 (−0.001, −0.00005)], *Gemmiger* [B (95% CI): −0.0003 (−0.001, −0.00009)] (Figure 7D), and *Fusicatenibacter* [B (95% CI): −0.0004 (−0.001, −0.0002)] (Figure 7B), and positively associated with *Escherichia_Shigella* [B (95% CI): 0.003 (0.001, 0.005)] (Figure 6E). BMI was significantly negatively associated with *[Ruminococcus]_gnavus_group* [B (95% CI): −0.002 (−0.003, −0.00005)] (Figure 7A). Sleep quality was significantly positively associated with *[Ruminococcus]_gnavus_group* [B (95% CI): 0.019 (0.004, 0.034)]. Smoking status showed no significant associations with any of the analyzed genera. Pesticide exposure was significantly negatively associated only with *Romboutsia* [B (95% CI): −0.017 (−0.023, −0.011)] (Figure 7F). Associations between other variables and the genera did not reach statistical significance (Figures 8A–E).

## 4 Discussion

This study aimed to investigate the factors influencing the gut microbiota of rural residents under different physical activity levels. Given that there was no difference in overall microbial diversity, and that the correlation analyses and multiple logistic regression analyses showed very small associations with the relative abundances of only a few genera, these findings should be interpreted as exploratory rather than as evidence of biologically meaningful differences.

Regarding microbial diversity, the MPA group exhibited the highest Shannon index, suggesting that moderate-intensity physical activity may be associated with a relatively healthy gut microecological structure. In contrast, although the LPA group had a higher Chao 1 index, its Shannon index was the lowest, indicating poor evenness of species distribution and consequently lower overall diversity. The HPA group showed relatively low levels across multiple indices, suggesting that excessively high-intensity physical activity may negatively affect gut microbiota diversity, which is consistent with previous studies (Suther et al., 2025). Beta diversity analysis revealed no significant separation in community structure. The gut microbiota is regulated by multiple factors including diet, genetics, age, and living environment (Gacesa et al., 2022); physical activity, as a single exposure factor, may have its independent effects masked by within-group variation of other confounding factors. Moreover, as a highly adaptable ecosystem, the core functional microbiota of the gut (e.g., major groups of *Bacillota* and *Bacteroidota*) may remain relatively stable across different physiological states to maintain basic metabolic and immune homeostasis (Pedroza Matute and Iyavoo, 2023).

The species composition analysis revealed that the MPA group exhibited a high abundance of Bacillota (particularly Lachnospiraceae and Ruminococcaceae), higher levels of *Faecalibacterium* and *Blautia*, and the lowest abundance of Enterobacteriaceae. This suggested that MPA conditions might be beneficial for maintaining gut homeostasis and short-chain fatty acid production. This suggests that MPA conditions may be conducive to maintaining intestinal homeostasis and short-chain fatty acid production. In contrast, the LPA group showed expansion of the Proteobacteria and an increase in *Escherichia_Shigella*, which are classic markers of gut dysbiosis. Proteobacteria is one of the most diverse bacterial phyla associated with many diseases. For example, certain pathogenic *Escherichia coli* strains (belonging to Proteobacteria) have been implicated in intestinal diseases such as inflammatory bowel disease (Hager et al., 2019). Enrichment of *Escherichia_Shigella* (15.4%) is closely associated with intestinal inflammation, impaired barrier function, and metabolic disorders (Markus et al., 2025). Although this group had a relatively high abundance of *Bifidobacterium*, which is generally considered a beneficial genus (Gavzy et al., 2023), the overall microbial structure shifted toward a pro-inflammatory direction, suggesting that the LPA group may have a negative impact on the intestinal barrier or immune environment. The microbiota of the HPA group, while better than that of the LPA group, did not reach the level of the MPA group. Studies have found that both low and high levels of physical activity are adversely associated with gut microbiota (Yuan et al., 2018; Baldanzi et al., 2024; Mao et al., 2024). Moderate physical activity may be more favorable for microecological balance. One study found that moderate-intensity sustained exercise generally promotes increased microbial diversity, an increase in short-chain fatty acid-producing bacteria, and enhanced intestinal barrier integrity. In contrast, excessive or high-intensity endurance exercise may lead to dysbiosis, inflammation, and increased intestinal permeability (Mohr et al., 2025). A balanced moderate-intensity exercise regimen combined with a healthy diet is the optimal strategy for enhancing gut microbiota diversity, reducing inflammation, and supporting overall health in athletes.

LEfSe analysis revealed that in the HPA group, *g_Veillonella*, *g_Solobacterium*, and *g_Weissella* were significantly enriched. *Veillonella* is a common commensal bacterium in the oral cavity and intestine, primarily involved in lactate metabolism (Zhang and Huang, 2023), which may be related to higher lactate levels in individuals with high physical activity. *Weissella* is a lactic acid bacterium in the gut that can modulate metabolism and exert neuroprotective effects (Hu et al., 2025). For example, *Weissella cibaria* alleviates Parkinson’s disease by regulating the gut microbiota, inhibiting the growth of pathogenic bacteria (e.g., *Proteus mirabilis*), reducing neuroinflammation (decreasing Tumor Necrosis Factor-alpha and Interleukin-1 beta expression in the brain and colon), and suppressing abnormal α-synuclein aggregation (Choi et al., 2025). In contrast, *g_Solobacterium* is an anaerobic *Gram-positive rod* in the gut that can increase the secretion of pro-inflammatory cytokines by activating the Nuclear Factor-kappa B signaling pathway, thereby disrupting intestinal barrier function (Yu et al., 2024). In the MPA group, *g_Rikenellaceae_RC9_gut_group*, *g_Ruminococcus_gauvreauii_group*, and *g_Staphylococcus* were significantly enriched. *Rikenellaceae_RC9_gut_group* is a dominant genus in the gut that regulates lipid metabolism, is crucial for fat breakdown, and participates in the nitrogen cycle, thus promoting gut microbiota diversity (Sebastià et al., 2023). *Ruminococcus_gauvreauii_group* ferments dietary fiber to produce short-chain fatty acids, inhibiting the growth of harmful bacteria. Moreover, this genus may reduce the risk of cardiovascular disease (Toya et al., 2020). Notably, *Staphylococcus* mainly comprises commensal or pathogenic species; for example, *Staphylococcus aureus* produces enterotoxins causing food poisoning, while *Staphylococcus epidermidis* protects the host against pathogen invasion (Wang et al., 2024). In the LPA group, enriched genera included *g_Hydrogenoanaerobacterium*, *g_Geobacillus*, *g_GCA 900066755 (unclassified)*, *g_Merdibacter*, and *f_Staphylococcaceae*, most of which are derived from environmental sources such as hot springs and soil (Braun et al., 2024). This may be related to their occupational environment. *Staphylococcus aureus*, a member of the family *Staphylococcaceae*, is a common pathogen that produces enterotoxins causing food poisoning, as well as skin infections (e.g., impetigo) and pneumonia (Larkin et al., 2009; Cieza et al., 2024). In summary, the composition and function of the gut microbiota are not only influenced by environmental factors but are also closely associated with host health status.

Through correlation analysis of the relationships between independent variables (e.g., physical activity level, sex, BMI) and gut microbiota, we found that age was the most significant factor influencing the gut microbiota. The trends of beneficial bacteria (e.g., *Bifidobacterium*, *Faecalibacterium*) decreasing with age and opportunistic pathogens (e.g., *Escherichia_Shigella*) increasing with age are consistent with previous studies. For example, a systematic study of individuals aged 0–70 years showed that *Bifidobacterium* levels strongly decreased with age, and the species composition of *Bifidobacterium* exhibited age dependence: *B. longum* predominated in infancy, *B. adolescentis* in adulthood, with further reductions in old age (Firrman et al., 2024). Additionally, another animal model study confirmed that the self-recovery capacity of the gut microbiota significantly declines in aged mice, and dysbiosis may accelerate the aging process (Shao et al., 2025). Physical activity level may be associated with specific genera. The observed reduction of *Blautia* and *Ruminococcus gnavus* in individuals with moderate-to-high physical activity levels has been reported in both athlete studies and animal models. For instance, a study comparing elite runners with healthy controls found that the relative abundance of *Blautia* was significantly lower in the athletes’ gut microbiota than in healthy controls (Dziewiecka et al., 2022), consistent with the negative correlation trend observed in our study. That study also found that microbial diversity and metabolic capacity were significantly higher in athletes than in controls (Dziewiecka et al., 2022), suggesting that the effects of exercise on the microbiota are selective. Animal model studies further confirmed that endurance-trained mice exhibited significantly lower relative abundance of *Ruminococcus gnavus* compared to sedentary controls, which aligns with our findings (Fernández et al., 2021).

Regarding the enrichment of *Segatella* in individuals with moderate-to-high physical activity levels, existing studies have found that this may be related to a tendency toward high-fiber diets in such populations, as this genus is typically more abundant in plant-based diets, possibly associated with the dietary preferences of rural residents (Fernández et al., 2021). We also found that *Bifidobacterium* abundance was higher in females, while *Collinsella* abundance was higher in males. The mechanisms underlying sex-related differences in gut microbiota are not fully understood, but current research suggests that sex hormones may shape microbial structure by influencing the intestinal environment (e.g., bile acid metabolism, immune regulation) (Tang et al., 2025). We further observed that *Escherichia_Shigella* was positively correlated with BMI. Multiple studies have confirmed that *Escherichia_Shigella* is elevated in obese and metabolically disordered populations; this genus has pro-inflammatory properties and may contribute to obesity-related metabolic abnormalities by inducing low-grade inflammation (Castaño-Rodríguez et al., 2018).

Additionally, *Romboutsia* and *Roseburia* were significantly reduced in individuals with pesticide exposure. A study on heavy metal arsenic exposure found that *Roseburia intestinalis* was significantly reduced in arsenic-exposed mice, and supplementation with this bacterium alleviated arsenic-induced intestinal damage, indicating a protective role of *Roseburia* against environmental toxicant exposure (Zhou et al., 2025). The negative correlation between *Roseburia* and pesticide exposure found in our study is consistent with that report. However, although a statistically significant negative correlation was observed, the effect size was very small. Notably, *Faecalibacterium* showed a significant negative correlation with sleep quality. A meta-analysis of 15 case–control studies found that the abundances of *Faecalibacterium* and *Lachnospira* were reduced in patients with insomnia, and insomnia severity was negatively correlated with *Faecalibacterium* abundance (Wang et al., 2025). This provides strong support for the association between *Faecalibacterium* and sleep quality observed in our study. Furthermore, we found that smokers had decreased *Bifidobacterium* abundance and increased *Collinsella* abundance, while drinkers had increased abundances of *Blautia*, *Collinsella*, and *[Ruminococcus]_gnavus_group*. The inhibitory effect of smoking on *Bifidobacterium* has been confirmed in multiple studies (Tomoda et al., 2011; Hu et al., 2015; Savin et al., 2018), possibly related to the direct toxic effects of harmful substances in tobacco on the gut microbiota. One study found that *Blautia* abundance was significantly elevated in patients with irritable bowel syndrome (IBS) and was positively correlated with body fat percentage exceeding 40% (OR = 2.85), suggesting that *Blautia* may play a role in intestinal dysfunction and metabolic abnormalities (Pecyna et al., 2025). The elevation of *Blautia* in drinkers may be associated with alcohol consumption and intestinal inflammation.

Given the substantial differences in gut microbiota between males and females, we further performed sex-stratified analyses to explore whether sex influences the relationship between physical activity level and gut microbiota. The results are presented in Table 4. In the male group, physical activity level was significantly negatively correlated with *Blautia* and *[Ruminococcus]_gnavus_group*; as physical activity increased, the beneficial bacteria *Blautia* and *[Ruminococcus]_gnavus_group* significantly decreased. A significant positive correlation was observed with *Segatella*; as physical activity increased, *Segatella* significantly increased. In contrast, in the female group, physical activity level showed no significant correlations with any of the analyzed genera. One study confirmed a significant association between *Ruminococcus gnavus* and male health indicators (including physical activity-related muscle mass) in a human cohort, whereas no such association was found in females (Park et al., 2024). Our findings are consistent with that study. However, the distribution of sex across physical activity groups was unbalanced; in particular, the low physical activity (LPA) group had a small sample size and consisted almost entirely of males, which precluded robust inference regarding the independent effect of low physical activity. The findings related to LPA in this study were therefore essentially limited to males.

**Table 4:** Correlation coefficients between sex and gut microbiota.

Genus	Male (*N* = 172)		Female (*N* = 139)	
	*r*	*p*-value	*r*	*p*-value
*Unclassified*	0.137	0.072	−0.090	0.294
*Escherichia_Shigella*	0.100	0.192	−0.045	0.598
*Blautia*	−0.224	0.003	−0.042	0.621
*Faecalibacterium*	0.112	0.144	0.069	0.417
*Bifidobacterium*	−0.144	0.059	−0.022	0.796
*Bacteroides*	−0.084	0.272	−0.081	0.341
*Megamonas*	0.021	0.782	0.043	0.619
*Segatella*	0.247	0.001	0.023	0.792
*Collinsella*	−0.103	0.179	0.064	0.457
*Agathobacter*	0.102	0.184	−0.013	0.883
*[Ruminococcus]_gnavus_group*	−0.180	0.018	−0.023	0.787
*Ruminococcus*	0.041	0.597	0.061	0.474
*Mediterraneibacter*	0.014	0.853	−0.033	0.703
*Anaerobutyricum*	−0.034	0.655	0.005	0.951
*Streptococcus*	−0.068	0.372	0.057	0.506
*Gemmiger*	0.073	0.344	0.071	0.403
*Roseburia*	0.073	0.345	0.050	0.561
*Fusicatenibacter*	−0.047	0.543	0.050	0.060
*Romboutsia*	−0.070	0.363	0.072	0.401
*Anaerostipes*	−0.039	0.609	0.027	0.751

We further examined the interaction between sex and physical activity. However, the interaction test results showed that sex differences in the effect of physical activity were only observed for the abundance of *Segatella*, whereas the effects on *Blautia* and *[Ruminococcus]_gnavus_group* were similar between males and females, with no significant interaction. A study using large-scale metagenomic meta-analysis found that members of the *Segatella copri* complex exhibit a reproducible and significant association with male sex. That study expanded the originally recognized single species into 13 distinct species and revealed a significant sex bias in their distribution in the human gut (Blanco-Míguez et al., 2023), providing strong support for our findings. Notably, although we adjusted for physical activity level, we were unable to distinguish between types of exercise. Future studies should collect more detailed parameters of physical activity.

As a comprehensive indicator of an individual’s physiological reserve, physical function is closely associated with physical activity level. Therefore, we analyzed the correlation between physical function and physical activity level. The results showed that muscle strength was significantly negatively correlated with physical activity level. This may be because the physical activity of rural residents is predominantly agricultural labor, including prolonged bending, heavy lifting, and repetitive movements, which are not equivalent to structured resistance training. Individuals engaged in long-term agricultural labor may not necessarily have higher muscle strength than those with light physical activity; instead, they may experience decreased muscle strength due to factors such as chronic fatigue accumulation and joint wear (Hyvärinen et al., 2025). A study of rural residents in Rwanda showed that body weight was significantly negatively correlated with lumbar extensor endurance (*R* = −0.60, *p* = 0.031), indicating that long-term load-bearing labor may lead to fatigue accumulation and decreased muscle strength (Sibson et al., 2024). Reaction time was significantly positively correlated with physical activity level, meaning that higher physical activity levels were associated with slower reaction speed. One study showed that when physical exertion was performed concurrently with a sustained attention task, the central nervous system’s ability to activate muscles was impaired, manifested as a decrease in maximum voluntary activation of approximately 6% (*p* < 0.001) and a 15.7–26.2% reduction in cognitive task accuracy (Goepp et al., 2024). The daily labor of rural residents, especially middle-aged and older adults, often involves repetitive, monotonous movements that require maintaining fixed muscle contraction patterns over long periods, which can lead to the accumulation of neuromuscular fatigue. Furthermore, the additive effect of age and physical activity level may also contribute to the decline in reaction speed. Balance ability showed no significant correlation with physical activity level. Balance with eyes closed primarily reflects proprioceptive and vestibular function rather than pure muscle strength (Hill et al., 2020).

Multiple linear regression analysis showed that after adjusting for multiple covariates, physical activity level was independently and significantly positively associated only with the genus *Segatella*. This is consistent with previous studies. However, although a statistically significant negative correlation was observed, the effect size was very small. *Prevotella* and *Segatella* are important keystone genera in the gut microbiota, renowned for their remarkable fiber-degrading capacity (Wang et al., 2026). They are enriched in physically active individuals, and their abundance is positively correlated with training duration (Cataldi et al., 2022). The abundance of these genera in the gut microbiota of athletes is significantly higher than that in sedentary individuals (Cataldi et al., 2022). Furthermore, a cross-sectional study found that middle-aged women with lower physical activity levels had significantly reduced abundance of this genus (Zheng et al., 2024), suggesting a positive association with physical activity level.

After adjusting for covariates including physical activity, BMI, and smoking and drinking status, sex was independently and significantly associated with the genera *Bacteroides* and *Collinsella*. The present study found that females had higher abundance of *Bacteroides* and lower abundance of *Collinsella*. The influence of sex on gut microbiota has been confirmed in multiple studies, although the effect size often varies across populations. A study in a C57BL/6 mouse model found that the relative colonization level of *Bacteroides* in the gut was significantly higher in female mice than in males, and sex influenced the regulatory effect of this genus on host immune responses (Valls et al., 2025). Using 16S rRNA sequencing analysis, that study revealed that the relative abundance of *Bacteroides* was significantly lower in male mice than in females. Our findings are consistent with previous research. This sex-dependent difference may be attributed to the modulation of the intestinal microenvironment by sex hormones.

After adjusting for multiple covariates, age was independently and significantly associated with several genera, exhibiting a regular pattern of age-related change. However, the effect sizes were all very small. Genera such as *Blautia*, *Anaerostipes*, *Faecalibacterium*, *Mediterraneibacter*, *Collinsella*, *Gemmiger*, and *Fusicatenibacter* decreased with age, while *Escherichia_Shigella* showed an increasing trend. A study of healthy controls, patients with colorectal adenoma, and patients with colorectal cancer demonstrated that the abundance of butyrate-producing bacteria was significantly higher in young healthy subjects than in elderly subjects. That study also noted that lactate-producing and butyrate-producing bacteria were more abundant in the healthy control group, suggesting that these bacteria may play an important role in preventing the progression of colorectal cancer (Song et al., 2023). Furthermore, a study of the gut microbiome in patients with acute gastroenteritis found that patient age was associated with overall microbial composition; elderly patients exhibited decreased abundance of *Faecalibacterium* and a pro-inflammatory enterotype dominated by *Escherichia-Shigella* (Castaño-Rodríguez et al., 2018). An analysis of the gut metagenome of Asian octogenarians also revealed decreased abundances of genera such as *Blautia*, *Collinsella*, *Faecalibacterium*, and *Mediterraneibacter* in the elderly population (Ravikrishnan et al., 2024). Notably, both *Faecalibacterium* and *Anaerostipes* are important butyrate-producing bacteria (Van-Wehle and Vital, 2024); their decreased abundance is consistent with age-related gut microecological decline. In contrast, the enrichment of *Escherichia_Shigella* in older individuals may reflect a trend of impaired intestinal barrier function and increased inflammatory levels.

After adjusting for multiple covariates, BMI was independently and significantly negatively associated with the genus *[Ruminococcus]_gnavus_group*. A meta-analysis encompassing 1,351 fecal metagenomic samples also confirmed that *Ruminococcus gnavus* is one of 38 bacterial species significantly altered in obesity (Hu et al., 2024). Our findings are consistent with the above report, further supporting that *Ruminococcus gnavus* may serve as a protective marker of metabolic health. The present study further found that sleep quality was significantly positively associated with the genus *[Ruminococcus]_gnavus_group*, suggesting that improved sleep may confer metabolic benefits by promoting the growth of beneficial bacteria. A cross-sectional study also indirectly supported the multidimensional association between *Ruminococcus gnavus* and metabolic health (Grahnemo et al., 2022).

After controlling for other covariates, smoking status in this study showed no significant association with any of the analyzed genera. Drinking status was independently and significantly negatively associated with the genus *Collinsella*. A study of the fecal microbiota in individuals with alcohol dependence directly confirmed that the relative abundance of *Collinsella* in the gut of alcohol-dependent individuals was significantly higher than that in non-drinking controls (Gao, 2024; Tsuruya et al., 2016). Our findings are consistent with the above literature. Pesticide exposure was significantly negatively associated only with the genus *Romboutsia*, indicating that environmental pollutants have a strong inhibitory effect on this genus. A study found that non-dietary exposure is an important route of pesticide accumulation in agricultural workers, and the abundance of *Romboutsia* was significantly lower in agricultural populations than in non-agricultural populations (Tosti et al., 2025). This result suggests that *Romboutsia* may serve as a sensitive biomarker of pesticide exposure, and changes in its abundance may reflect the health risk of pesticide exposure in rural residents. However, because our questionnaire did not collect detailed information on pesticide use (e.g., type, frequency, duration, protective measures), we were unable to perform a more precise exposure assessment. Therefore, this finding should be considered fragile and interpreted with caution. Future studies should employ more accurate exposure assessment methods (e.g., biomonitoring or detailed usage records) to validate this association.

The limitations of this study are as follows. First, the cross-sectional nature of this study does not prove causal relationships or long-term effects between different physical activity levels, BMI, age, drinking status, smoking status, pesticide exposure, sleep quality, exercise status, and gut microbiota. The findings of this study were considered exploratory and hypothesis-generating and could not be used as a basis for causal inference. Second, our study only included Chinese rural residents; the influence of different ethnicities and countries still needs to be considered. It has been reported that the composition of the gut microbiota differs across ethnicities and countries (Andreu-Sánchez et al., 2025). Therefore, caution is needed when generalizing our findings. Third, the multiple regression analysis in this study did not include gastrointestinal diseases, dietary fiber intake, dietary habits, type of labor, occupational environment, probiotic intake, or supplement use as covariates, and these factors may have an impact. Even after adjusting for all such factors in regression analysis, residual confounding may still exist. Fourth, in the multiple regression analysis of this study, the associations between gut microbiota and different physical activity levels, BMI, age, drinking status, smoking status, pesticide exposure, sleep quality, and exercise status all showed relatively small *B*-values. These findings might have been influenced by confounding factors or the sample size. Given the small effect sizes, these findings should be interpreted as exploratory and hypothesis-generating rather than as evidence of biologically meaningful differences. Notably, the relatively small sample size in our study may have led to unstable *B* values. Therefore, future studies with larger sample sizes are needed to provide more robust conclusions. Fifth, the assessment of physical activity level in this study was mainly based on questionnaires, which may not accurately reflect the cumulative effects of long-term, high-intensity exercise on the microbiota. Sixth, changes in the microbiota may exhibit individual differences or lag effects in response to physical activity levels, and the cross-sectional design makes it difficult to capture such dynamic relationships. Future longitudinal intervention studies or more precise accelerometer measurements would help to further clarify the potential effects of physical activity level on the gut microbiota. Seventh, factors such as age, BMI, type of labor, and dietary structure are also important influences on the association between physical activity and gut microbiota. However, due to uneven sample size distribution, stratification would reduce the sample size in each stratum, and too few stratified samples would decrease the stability of the analysis results. Therefore, we performed stratified analysis only for sex and not for other variables. Eighth, no correction for multiple comparisons was performed in this study. Findings with *p*-values near 0.05 therefore had a relatively high probability of being false positives. We strongly advised readers not to interpret these nominally significant associations as definitive conclusions, but rather as preliminary clues requiring further validation. Future studies should adopt more stringent statistical corrections and perform validation in independent, larger-sample cohorts. Ninth, diet is known to be the most dominant driver shaping gut microbiota composition; however, detailed dietary intake data were not collected in this study. A large body of literature has demonstrated that dietary fiber, fat, protein, and specific food components are among the strongest predictors of gut microbiota composition. Because a pilot study revealed that compliance with a long-form food frequency questionnaire was extremely low in this rural population, and recall bias was severe, we ultimately decided not to include a detailed dietary survey. Consequently, we could not rule out the possibility that the weak associations observed between physical activity and gut microbiota were wholly or partially confounded by unmeasured dietary factors. We strongly advised readers to regard the findings of this study as exploratory. Future studies should employ more reliable dietary assessment tools supplemented with objective biomarkers, and validation in independent cohorts with rigorous dietary evaluation is necessary.

Nevertheless, our study is innovative. Most existing research on the gut microbiota has focused on urban populations or patients with specific diseases, while large-scale cross-sectional studies targeting rural residents remain relatively scarce. Given that rural residents typically have unique dietary patterns, higher exposure to environmental microorganisms, and more traditional modes of production, their gut microbiota characteristics and influencing factors may differ substantially from those of urban populations. By specifically selecting rural residents as the study population, this research systematically characterizes the gut microbiota of this special population across different physical activity levels, thereby addressing gaps in population coverage and gut microecological diversity in existing studies. This perspective is highly timely. Overall, this study provides a new perspective for the health management of rural residents and offers a scientific basis for the future development of precise health intervention strategies and chronic disease prevention strategies tailored to rural residents with different activity patterns.

## 5 Conclusion

In summary, the results of this study indicated that there were no differences in gut microbiota diversity across different physical activity levels, and only specific genera were associated with physical activity level. Moderate-intensity physical activity was found to promote the enrichment of short-chain fatty acid-producing bacteria. Physical activity level was independently and positively associated only with the genus *Segatella*, and its modulation of genera such as *Blautia* and *[Ruminococcus] gnavus* exhibited sex-modifying effects. Age was the strongest independent factor influencing the gut microbiota, characterized by an age-related decline in multiple butyrate-producing genera and an increase in opportunistic pathogens. BMI, alcohol consumption, and pesticide exposure also had independent effects on specific genera. This study provides new insights from a large-sample investigation of the gut microbiota of rural residents in southern China and offers a scientific basis for the future development of precise health intervention strategies and chronic disease prevention strategies tailored to rural residents with different activity patterns. Future longitudinal intervention studies, larger multicenter studies incorporating confounding factors such as dietary habits, dietary fiber intake, and type of labor, accelerometer-based fine measurement, and integration of metagenomics or metabolomics to elucidate the functional pathways of key genera and their regulatory mechanisms on host metabolism and immunity are needed to further validate causal relationships and improve the robustness of the conclusions.

## Data Availability

The questionnaire data and the 16S rRNA data from this study have been deposited in Mendeley Data. The dataset can be accessed at: https://doi.org/10.17632/hrvjzn94nn.1.
